# ASO Author Reflections: Estimating the Prevalence of Pseudomyxoma Peritonei in Europe Using a Novel Statistical Method

**DOI:** 10.1245/s10434-020-08691-4

**Published:** 2020-06-03

**Authors:** Kjersti Flatmark, Faheez Mohamed

**Affiliations:** 1 Department of Gastroenterological Surgery, The Norwegian Radium Hospital, Oslo University Hospital, Oslo, Norway; 2 Department of Tumor Biology, The Norwegian Radium Hospital, Oslo University Hospital Oslo, Oslo, Norway; 3 Faculty of Medicine, University of Oslo, Oslo, Norway; 4 Peritoneal Malignancy Institute, Basingstoke and North Hampshire Hospital, Basingstoke, UK

## Past

Pseudomyxoma peritonei (PMP) is an uncommon abdominal cancer characterised by extensive growth of mucinous tumour in the peritoneal cavity. Historically, surgical debulking has been the mainstay of treatment. From the 1980s complete cytoreductive surgery with hyperthermic intraperitoneal chemotherapy (HIPEC) has been developed as a curative treatment strategy, but this approach is resource intensive with the best outcomes in high volume centres^1^^,^^2^. Lack of reliable epidemiological data has hampered adequate diagnosis and treatment of PMP globally.

## Present

Based on analysis of data from Norway and England, a minimum incidence rate of 3.2 per million and a prevalence rate of 22 per million per year was determined for PMP,^4^ which is higher than previously suggested.^3^ Extrapolating this, using a novel statistical method, we estimated that 11,736 people in Europe were alive with a diagnosis of PMP in 2018.^4^

## Future

This work estimates the burden of disease caused by PMP in Europe. Through organisations such as the EuroPMP COST Action (https://europmp.eu; funded by COST—European Cooperation in Science and Technology) we are working to raise awareness and improve management of PMP by establishing networks to promote education, training and research across Europe. With early diagnosis, referral to a centre of expertise, judicious use of cytoreductive surgery with hyperthermic intraperitoneal chemotherapy, and appropriate surveillance, these patients can enjoy a good quality of life with the chance of cure from what can otherwise be a debilitating and fatal disease.^5^^,^^6^ We hope that the analysis presented in this article^4^ will encourage the establishment of prospective registries to inform healthcare providers and ensure appropriate resource allocation so that optimal care may be offered to all patients with PMP and other rare cancers.
